# Regulation of c-Jun NH_2_-Terminal Kinase for Islet Transplantation

**DOI:** 10.3390/jcm8111763

**Published:** 2019-10-23

**Authors:** Hirofumi Noguchi

**Affiliations:** Department of Regenerative Medicine, Graduate School of Medicine, University of the Ryukyus, Okinawa 903-0215, Japan

**Keywords:** islet transplantation, c-Jun NH_2_-terminal kinase (JNK), mitogen-activated protein kinases (MAPKs), pancreas preservation, islet isolation, apoptosis

## Abstract

Islet transplantation has been demonstrated to provide superior glycemic control with reduced glucose lability and hypoglycemic events compared with standard insulin therapy. However, the insulin independence rate after islet transplantation from one donor pancreas has remained low. The low frequency of islet grafting is dependent on poor islet recovery from donors and early islet loss during the first hours following grafting. The reduction in islet mass during pancreas preservation, islet isolation, and islet transplantation leads to β-cell death by apoptosis and the prerecruitment of intracellular death signaling pathways, such as c-Jun NH_2_-terminal kinase (JNK), which is one of the stress groups of mitogen-activated protein kinases (MAPKs). In this review, we show some of the most recent contributions to the advancement of knowledge of the JNK pathway and several possibilities for the treatment of diabetes using JNK inhibitors.

## 1. Introduction

Type 1 diabetes mellitus (T1DM) is an autoimmune disease and usually diagnosed at a young age with insulin deficiency. T1DM is characterized by progressive β-cell failure and gradual destruction of β-cells [1]. According to the International Diabetes Federation, approximately 542,000 children 0–14 years of age have T1DM, with 86,000 new cases diagnosed worldwide each year [1]. In the insulitis lesion in T1DM, invading immune cells produce cytokines, such as interleukin (IL)-1β, tumor necrosis factor (TNF)-α, and interferon (IFN)-γ [2]. IL-1β, TNF-α, and IFN-γ induce β-cell apoptosis via the activation of β-cell gene networks under the control of the transcription factors nuclear factor-κB (NF-κB) and STAT-1. NF-κB activation leads to the production of nitric oxide (NO) and chemokines and the depletion of endoplasmic reticulum (ER) calcium [3,4,5]. The execution of β-cell death occurs through the activation of mitogen-activated protein kinases (MAPKs), via the triggering of ER stress and the release of mitochondrial death signals.

**Figure 1:**
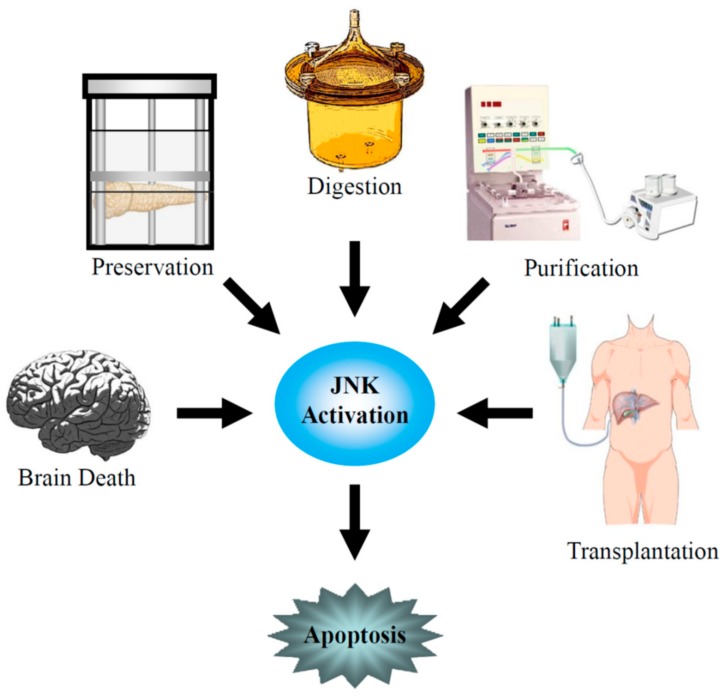
c-Jun NH_2_-terminal kinase (JNK) activation during brain death and pancreas procurement, pancreas preservation, islet isolation, and islet transplantation.

Pancreatic islet transplantation has recently emerged as one of the most promising therapeutic approaches to improving glycometabolic control in T1DM patients and, in many cases, achieving insulin independence. Application of the Edmonton protocol has markedly improved the outcome [6,7] and the rate of insulin independence after islet transplantation has significantly improved in recent years [8]. However, multiple islet infusions from two or more donors are often required to achieve and maintain insulin independence. Contributions to graft loss include the instant blood-mediated inflammatory reaction (IBMIR), potent host auto- and allo-immune responses, and β-cell toxicity from immunosuppressive agents [9,10,11,12]. Moreover, the isolation procedure of pancreatic islets itself destroys cellular and noncellular components of the pancreatic tissue, which presumably play a role in supporting the survival of islet cells [13,14]. Pancreatic islets are exposed to mechanical and warm/cold ischemic stresses during pancreas procurement; to osmotic and cold ischemic stresses during pancreas preservation; to mechanical, enzymatic, and warm ischemic stresses during pancreas digestion; and to mechanical, osmotic, and cold ischemic stresses during islet purification. These stresses induce β-cell death (Figure 1).

β-cell death by apoptosis and the prerecruitment of intracellular death signaling pathways immediately after isolation and transplantation contributes to a reduction of the islet mass [15,16,17]. NF-κB [16] and the stress-associated MAPKs [18,19] mainly act as death-signaling pathways and these factors have been shown to contribute to the apoptosis of pancreatic β-cells. Inhibition of the death-signaling pathways has proven to be beneficial in several models of insulin-producing cell apoptosis in vitro [20,21]. Three major conserved groups of stress-associated MAPKs have been described: p38 kinases (p38 α/β/γ/δ) [22], c-Jun NH_2_-terminal kinases (JNKs; JNK1/2/3) [23], and extracellular signal-regulated kinases (ERKs; ERK1/2/3) [24]. JNK and p38 are similarly activated by several stresses, such as cold and heat shock, hypo- and hyperosmolarity, shearing stresses, proinflammatory cytokines, cytotoxic drugs, ultraviolet and γ-irradiation, the loss of survival factors, and reactive oxygen species [25,26]. Both p38 and JNK activate downstream nuclear transcription factors, which participate in the cellular response [27,28] by, for example, activating transcription factor-2 (ATF-2) and the activator protein-1 (AP-1), which is formed of heteromers of c-fos and c-Jun [27,28,29,30].

Here, we review the advancement of knowledge on the death-signaling pathways, especially the JNK pathway, during pancreas preservation, islet isolation, and islet transplantation, and the effect of JNK inhibitors for islet transplantation.

## 2. Donor Organ

The cadaver donor is the principal source of organs for transplantation. However, the successful rate of transplantations, such as those of kidneys, both over the short- and long-term, remains significantly inferior to those from living donors [31]. The differences between cadaveric and living donors are brain death, an optimal health condition of the living donor, a marginal condition of a substantial number of cadaveric donors, and an optimal timing of surgery in the case of living donors in comparison to a long cold ischemia time in cadaveric donors. It is certified that brain death affects the hemodynamic status, inflammatory reactivity, and hormone regulation. The effect of massive acute cerebral injury, as well as hypotension and circulating factors, results in the deterioration of organs following brain death [32,33]. Brain death is characterized by extensive cortical necrosis, which stimulates multiple cell types to produce proinflammatory cytokines, including IL-1β, TNF-α, IFN-γ, and IL-6 [32,33,34,35,36,37]. In the pancreas, donor characteristics such as age, cause of death, length of hospitalization, and medical history have a remarkable impact on islet recovery after isolation [38]. It has been demonstrated that the release of these proinflammatory cytokines, associated with brain death, significantly reduces the islet yield, functionality, viability, and engraftment after transplantation [36]. In this context, islets recovered from brain death donors presented higher nuclear activity of inflammation-related transcription factors, including ATF-2, c-Jun, and NF-κB. Furthermore, it has been demonstrated that macrophages infiltrate islets during brain death and that macrophage-associated inflammatory molecules, such as IL-1β, TNF-α, and IL-6, in islets are induced by brain death [39]. Therefore, the establishment of therapeutic strategies to prevent the deterioration of pancreatic islets during brain death could improve the islet transplant outcome. In addition, the strategies could improve the quality of organs from marginal donors, thus broadening the criteria for donor acceptance for isolation and transplantation.

It has been reported that males are more susceptible to the life-threatening effects of sepsis, hemorrhage, and trauma, compared to females in the proestrus cycle [40,41]. Female sex steroids, such as 17β-estradiol and estrogen, are likely to exhibit protective properties of immune and cardiovascular function after trauma, severe blood loss, and various adverse conditions [40,42]. Estradiol administration reversed the spontaneous increase of proinflammatory cytokines, such as TNF-α, IL-1β, and IL-6 [43]. Moreover, estrogen possesses significant antiapoptotic and antioxidant activities [42,43]. Eckhoff et al. reported that 17β-estradiol treatment significantly decreased proinflammatory cytokine and structural and physiologic derangements in pancreatic islets subsequent to brain death induction [44]. In addition, it was demonstrated that estradiol improves the survival and functionality of human islets after proinflammatory cytokine exposure in vitro and in vivo. The molecular mechanisms involved included the inhibition of JNK activation, NF-κB nuclear translocation, caspase-9 activation, and mitochondrial cytochrome c release [45,46]. The inhibition of JNK activation induced the reduction of JNK targets, including the nuclear activities of transcription factors ATF-2, AP-1, c-Fos, c-Jun, and Jun-D, involved in apoptosis in pancreatic β-cells [46].

Our group investigated whether the administration of JNK inhibitors in human and porcine pancreata immediately after the procurements improves islet isolation results by preventing the apoptosis of islet cells [47]. A low molecular weight JNK inhibitor (SP600125) and a cell-permeable JNK inhibitor were used in porcine and human studies, respectively. The administration of JNK inhibitors in both porcine and human pancreata prevented JNK activation during the isolation procedure and prevented islet apoptosis immediately after isolation. Our data demonstrated that the JNK pathway is the major mediator of islet deterioration during/immediately after isolation and that JNK inhibition before islet isolation could improve the outcomes after pancreatic islet transplantation. The treatment of multiorgan donors with JNK inhibitors or 17β-estradiol could improve the quality of organs from marginal donors and increase human islet yields and functionality, and therefore broaden the criteria for donor acceptance for islet isolation and transplantation.

## 3. Pancreas Preservation

During pancreas preservation, islets are exposed to serious damaging conditions, resulting in a reduction of islet survival and ultimately graft failure after transplantation. The University of Wisconsin (UW) solution has been recognized as the gold standard in pancreas preservation before islet isolation. We, and other groups, have reported the superiority of the two-layer preservation method (TLM), which employs oxygenated perfluorochemical (PFC) and UW solution, compared with simple cold storage in UW for not only the whole pancreas, but also pancreatic islet transplantation in humans [48,49,50,51]. When TLM is used for pancreas preservation, PFC directly oxygenates the pancreas and results in a high level of adenosine triphosphate (ATP) in pancreatic tissues, which maintains parenchymal and nonparenchymal viability and retains cellular integrity [52,53,54,55]. Matsuda et al. reported the apoptosis pathways of caspase 3, 8, 9, JNK, and p38 in isolated islets after the cold storage of UW solution or TLM [56]. Islet apoptosis in the UW group was significantly increased compared with the fresh (no preservation) and TLM groups. Both caspase 3 and 9 activities in the UW group were higher than in the fresh and TLM groups, with an approximate increase of 2- to 3-fold. On the other hand, there was no significant difference in caspase 8 activity among these three groups. These data suggest that the mitochondrial pathway is largely engaged in islet apoptosis induced by the simple preservation of UW solution, and that TLM blocks to a great extent. On the other hand, JNKs were strongly activated in both the TLM and UW groups, while they were not activated in the fresh group. In contrast, p38 was activated to almost the same levels in these three groups. These findings suggest that pancreas preservation with UW solution or TLM before islet isolation cannot protect against JNK activation.

Our group showed that an intraductal injection of JNK inhibitors before pancreas storage prevented JNK activation during the isolation procedure and improved islet graft survival in humans. [47]. Another group also reported that an intraductal injection of JNK inhibitor in porcine pancreata significantly suppressed mRNA expression levels of IL-1β, TNF-α, IFN-γ, IL-6, IL-8, and macrophage chemoattractant protein-1, as well as the concentration of IL-1β and IL-8, in the culture supernatant [57]. These data suggest that the inhibition of JNK activation during pancreas preservation improves the islet transplant outcome through the reduction of the inflammatory response.

We recently developed a novel preservation solution, the extracellular-type/JNK inhibitor-containing (EJ) solution, for porcine pancreas preservation [58]. After pancreas preservation in EJ solution, JNK activity was maintained at a relatively low level during islet isolation. The islet yield before and after purification was significantly higher in the EJ group than in the UW group or EJ-J (EJ solution without the JNK inhibitor) group. After islet transplantation into streptozotocin-induced diabetic mice, the attainability of post-transplantation normoglycemia was higher in the EJ group than the UW group or EJ-J group. These data suggest that the inhibition of JNK activity for pancreas storage could be useful for preventing islet apoptosis and improving islet transplant outcomes.

## 4. Islet Isolation and Culture

Pancreatic islets are exposed to mechanical, enzymatic, osmotic, and ischemic stresses during pancreas digestion and islet purification. Our group reported that JNK activity progressively increased during the isolation procedure [47]. Abdelli et al. mapped the major intracellular stress-signaling pathways activated during human islet isolation and following acute cytokine exposure [17]. For the islet isolation procedure, two pathways are involved in islet survival: NF-κB→iNOS and MAPK kinase 7 (MKK7)→JNK/p38→c-fos. Proinflammatory cytokines activate the NF-κB→iNOS and MKK4/MKK3/6→JNK/p38 pathways without the involvement of c-fos. It is also likely that the procedure of islet isolation, together with proinflammatory cytokine production immediately after transplantation, may further synergize to enhance the apoptosis of islets [59]. In the case of other cell types, MKK7 also transduces cytokine signaling [26]. Therefore, the activation of MKK7 after islet purification may sensitize islets to cytokine exposure [60]. The activated pathways return to background levels after the 48 h culture of isolated islets and the expression of MKK7 becomes undetectable [17]. Inhibition of the JNK, p38, and NF-κB pathways throughout the procedures of pancreas preservation, islet isolation, and islet transplantation might result in the reduction of primary nonfunction and the improvement of islet graft survival [20,21,61]. Our group also reported that the treatment of JNK inhibitors before islet isolation prevented JNK activation during the isolation procedure and prevented islet apoptosis immediately after isolation [47].

Three JNK isoforms (JNK1, 2, and 3) have been identified. JNK1 and JNK2 are ubiquitously expressed, while JNK3 expression is restricted to pancreatic islets and the brain [62,63]. In contrast to JNK1 and JNK2, JNK3 exhibits anti-apoptotic activity in insulin-producing cells [63]. Varona-Santos et al. investigated the role of JNK isoforms in pancreatic islets using *Jnk1^−/−^* and *Jnk2^−/−^* mice [64]. Islets derived from *Jnk1^−/−^* mice secreted more insulin and significantly protected cytokine-induced cell death compared with islets derived from wild-type and *Jnk2^−/−^* mice. These data suggest that specific JNK1 blockades in islets may be important for islet transplantation [64].

## 5. Islet Transplantation

The transplantation of isolated islets into the liver through the portal vein is the preferred site for clinical islet transplantation. An early innate inflammatory reaction after intrahepatic islet transplantation strongly affects islet engraftment and survival. This early immune response is triggered by ischemia-reperfusion injury and IBMIR occurring hours and days after islet infusion [65,66,67,68,69,70,71]. IBMIR involves activation of the complement and coagulation cascades, ultimately resulting in clot formation and infiltration of leukocytes into the islets, which leads to disruption of islet integrity and islet destruction [12]. Moreover, the nonspecific activation and dysfunction of intrahepatic endothelial cells after islet transplantation, which are characterized by the production of proinflammatory cytokines such as TNF-α, IL-1β, and IFN-γ, as well as the upregulation of the intracellular adhesion molecule (ICAM)-1, P-selectin, and NO, have been demonstrated [69,70,71,72,73,74,75]. These effects finally induce early graft loss. It has been reported that 25% of the transplanted islets were lost within the first few minutes after intraportal transplantation [76] and that the islet loss after transplantation into the portal vein is widely estimated to be higher (50%–60%) [77,78,79]. To prevent early graft loss, candidate drugs have been reported in clinical and experimental animal studies. Heparin is commonly used for clinical islet transplantation to reduce the impact of coagulation. Low molecular weight dextran sulfate (LMW-DS, MM 5000) is an alternative inhibitor of IBMIR [80,81,82,83,84]. An open randomized multicenter study showed that LMW-DS has a similar efficacy in inhibiting IBMIR to promote islet engraftment when compared with heparin [84]. Activated protein C (APC) is another potent inhibitor which exerts anticoagulant, anti-inflammatory, and antiapoptotic activities by acting directly on cells. It has been reported that the exogenous administration of APC significantly reduced the loss of functional islet mass after intraportal transplantation in diabetic mice [85]. APC is an important physiological anticoagulant generated from protein C by the action of thrombin-thrombomodulin on endothelial cells [86]. APC appears to regulate the inflammatory process in part by blocking the activity of the transcription factor NF-κB by preventing the generation of thrombin and by inhibiting the production of proinflammatory cytokines [86,87,88,89,90]. Our group showed that the double blockage of proinflammatory cytokines, IL-1β and TNF-α, improved the efficacy of clinical islet transplantation [91]. The blockage of TNF-α, eternacept, IL-1β, and anakinra was administered in three patients with type 1 diabetes before and during islet transplantation and all patients achieved insulin independence with normal HbA1c levels by a single infusion from one donor. Although this study used not only the antibody, but also thymoglobulin induction and sirolimus-free immunosuppression, the double blockage of IL-1β and TNF-α could contribute to the prevention of early graft loss.

To evaluate the intracellular stress-signaling pathways of JNK during the islet transplant process, our group measured JNK activity in the liver 1, 3, 6, and 24 h after mouse islet transplantation [92]. The JNK was activated until 1 h after islet transplantation and the activity became gradually higher until 24 h. The evidence has profound implications for IBMIR, the production of proinflammatory cytokine, and subsequent islet apoptosis. Our group also investigated the effect of an intraportal injection of pancreatic islets with JNK inhibitor. Isolated islets with JNK inhibitor were transplanted into diabetic mice through the portal vein and liver samples were collected before transplantation and 1, 3, 6, and 24 h after transplantation. The JNK activity in the liver was suppressed at a low level until 24 h after transplantation. Moreover, the intraportal injection of isolated islets with the JNK inhibitor improved islet graft survival [92]. These data suggest that control of the JNK pathway is extremely important in islet transplantation and that an intraportal injection of isolated islets with JNK inhibitor prevents the activation of JNK in the liver immediately after islet transplantation and improves the outcome for islet transplantation.

Varona-Santos et al. investigated the role of JNK isoforms in transplant recipients using *Jnk1^−/−^* and *Jnk2^−/−^* mice [64]. When islets derived from wild-type mice were transplanted into diabetic *Jnk1^−/−^*recipients, the median time to diabetes reversal was shorter than that for wild-type diabetic recipients. On the other hand, the median time to diabetes reversal in diabetic *Jnk2^−/−^* recipients was longer than that for wild-type diabetic recipients when islets derived from wild-type mice were transplanted into diabetic *Jnk2^−/−^* recipients. These data suggest that specific JNK1 blockades in recipients may be important for islet transplantation [64].

## 6. JNK Inhibitors

JNK inhibitors have been expected as drugs to improve islet transplant outcomes (Table 1). The widely used inhibitor of JNKs for research is SP600125 [93]. SP600125 is an ATP-competitive inhibitor and the IC_50_ values for JNK1 and JNK2 are both 40 nM, while that for JNK3 is 90 nM [94]. On the other hand, SP600125 is >300-fold selective over the related MAPKs, ERK1, and p38-2, and between 10-fold and 100-fold selective over another 14 protein kinases tested [94]. We and another group showed the efficacy of SP600125 during pancreas preservation for islet transplantation [47,57]. SP600125 prevented JNK activation during islet isolation and improved isle viability and the islet transplant outcome. However, the ATP-competitive inhibitor has several degrees of toxicity and lacks the required specificity because it inhibits the phosphorylation of all JNK substrates [95].

**Figure 2:**
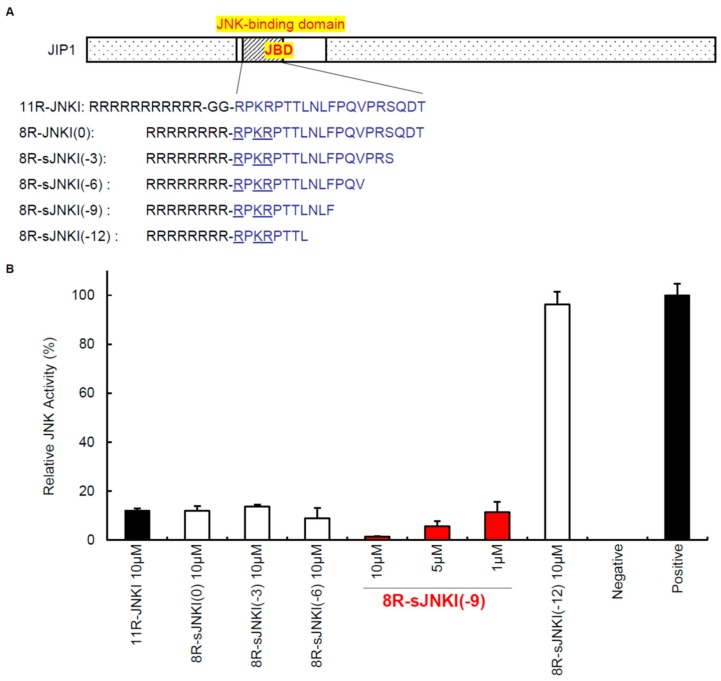
Cell-permeable JNK inhibitors. (**A**) The sequences of JNK inhibitory peptides. The peptide inhibitors of JNK are based on JNK-interacting protein-1 (JIP1), which was discovered to have a JNK inhibitory property. (**B**) Inhibition of JNK activation. MIN6 cells, which are part of the pancreatic β-cell line, were cultured with 1–10 μM of 11R-JNKI, 8R-JNKI(0), 8R-sJNKI(-3), 8R-sJNKI(-6), 8R-sJNKI (-9), or 8R-sJNKI(-12) for 23 h. The cells were then treated with 1 μg/mL of anisomycin for 1 h to stimulate the activation of JNK, after which the JNK activity was examined by western blotting. The cell lysates from MIN6 cells cultured with and without 1 μg/mL of anisomycin for 1 h were used as positive and negative controls, respectively. The data are expressed with the JNK activity of the positive and negative controls, which were arbitrarily set at 100 and 0, respectively.

Peptide inhibitors of JNK have also been developed, which are ATP-noncompetitive [93]. The peptide inhibitors of JNK are based on JNK-interacting protein-1 (JIP1), also known as islet-brain-1 (IB1). JIP1 has been discovered to have a JNK inhibitory property and its minimum inhibitory sequence has also been identified [20,96]. For efficient delivery of the JNK inhibitory peptide (JNKI) (Figure 2) into pancreatic islets, our group synthesized JNKI as a C-terminal fusion protein with 11-arginine (11R). Poly-arginine facilitates the uptake of peptides into mammalian cells more efficiently than TAT or other cell-penetrating peptides [97,98,99,100]. 11R-JNKI prevented JNK activation during pancreas preservation and islet isolation [47], islet culture [101], and immediately after islet transplantation [92], resulting in an improvement of islet graft survival. Another group also reported that TAT-JNKI reduced the islet loss in culture and protected against cell death through the regulation of AKT/GSK3B activity [102]. Our group recently developed a more efficient JNK inhibitory peptide [58,103]. The N-terminal amino acids of JNKI include two arginine and one lysin (RPKR) (Figure 2). Since poly-arginine/lysin facilitates the uptake of peptides and proteins into mammalian cells, our group hypothesized that the transduction efficacy of 11R-JNKI may not be reduced after the deletion of three arginine and two glycine-linkers. Moreover, we investigated whether C-terminal deletion peptides of JNKI can inhibit JNK activity (Figure 2). One of the peptides, 8R-sJNKI(-9), efficiently prevented JNK activation at one tenth of the concentration of 11R-JNKI, suggesting that 8R-sJNKI(-9) inhibits islet apoptosis and improves islet function more efficiently than 11R-JNKI. It has been reported that, when the specificity of sJNKI was investigated in assays of 40 different protein kinases, only the JNKs and their upstream activators MKK7 and MKK4 were affected, emphasizing the specificity of inhibition [104].

It has been reported that exenatide, a glucagon-like peptide-1 receptor agonist, inhibited JNK activation and caspase-3 activation, resulting in the inhibition of β-cell apoptosis [105] and that 17β-estoradiol reduced JNK activation, nuclear AP-1, c-fos, Jun-D, and ATF-2 activities and enhanced islet viability and islet mass [46]. Prolactin and α-1 antitrypsin also inhibited JNK activation [106,107,108].

## 7. Future Perspective

The activation of JNK is induced during pancreas preservation and JNK activity is progressively increased during the isolation procedure. In addition, JNK is activated in the transplanted liver immediately after islet transplantation. In diabetes, JNK plays an important role in various tissues due to the phenomenon known as “glucose toxicity” and activation of the JNK pathway interferes with insulin biosynthesis [109], β-cell function [109,110], and insulin action [111,112,113]. The JNK inhibition from pancreas preservation and isolation, and throughout the transplantation procedure might prove critical for the maintenance of islet cell mass and improve isle graft function. The current challenge in finding new successful anti-JNK therapies is to design isoform-selective inhibitors of the JNKs. The regulation of intracellular signaling pathways, including JNK, may become a new therapeutic strategy to improve graft survival in clinical islet transplantation.

Both JNK and p38 are preferentially activated in response to the processing of islets for transplantation and by the inflammation associated with islet transplantation. Some small molecules that inhibit p38 activity suppress the production of proinflammatory cytokines and improve islet engraftment [114,115,116]. Since p38 participates in another signaling cascade controlling cellular responses to cytokines and stress, the inhibition of both JNK and p38 may enhance the inhibition of isle apoptosis. Some groups indicate cytokine-mediated β-cell necrosis as an additional possibility [117,118]. Collier et al. showed that proinflammatory cytokines cause β-cell cytotoxicity primarily through a nonapoptotic mechanism linked to a decline in ATP levels [117]. Steer et al. showed that IL-1 induces β-cell necrosis [118]. The inhibition of not only apoptosis but also necrosis may become a new therapeutic strategy to improve the outcome of islet transplantation.

## Figures and Tables

**Table 1:** c-Jun NH_2_-terminal kinase (JNK) inhibitors used for islet transplantation.

Agents	Administration Step	Effect	Year	Reference
17β-estradiol	Brain death	Reduction in JNK activation, nuclear AP-1, c-fos, Jun-D, and ATF-2 activities Enhancement of islet viability and islet mass	2003	[46]
Cell-permeable peptide inhibitor (11R-JNKI)	Culture	Prevention of islet apoptosis Improvement of islet graft function	2005	[101]
Cell-permeable peptide inhibitor (11R-JNKI)	Transplantation	Prevention of islet graft loss Improvement of islet transplant outcome	2007	[92]
Cell-permeable TAT peptide inhibitor (L-JNKI)	Culture	Reduction of the islet loss in culture and protection from cell death regulation of AKT/GSK3B activity	2008	[102]
Cell-permeable peptide inhibitor (11R-JNKI), SP600125	Pancreas preservation	Prevention of JNK activation during the isolation procedure Improvement of islet transplant outcome	2009	[47]
SP600125 (+ simvastatin)	Pancreas preservation	Increase of the β-cell viability index and islet survival rate	2011	[57]
GLP-1 ^1^ receptor agonist (exenatide)	Culture	Lower JNK and caspase-3 activation and β-cell apoptosis	2013	[105]
α-1 antitrypsin	Transplantation	Suppression of JNK phosphorylation Suppression of blood-mediated coagulation pathways	2017	[106]
Prolactin	Culture	Prevention of the activation of JNK via AKT	2018	[107]
Cell-permeable peptide inhibitor (8R-sJNKI)	Culture	Prevention of islet apoptosis Improvement of islet graft function	2018	[103]
Cell-permeable peptide inhibitor (8R-sJNKI)	Pancreas preservation	Prevention of JNK activation during the isolation procedure Improvement of islet transplant outcome	2019	[58]
α-1 antitrypsin	i.p. injection 24 h before islet isolation	Suppression of JNK phosphorylation Suppression of caspase 9 activation	2019	[108]

^1^ GLP-1: Glucagon-like peptide-1.

## Abbreviations

JNK	c-Jun NH_2_-terminal kinase
MAPKs	mitogen-activated protein kinases
T1DM	type 1 diabetes mellitus
IL	interleukin
TNF	tumor necrosis factor
IFN	interferon
NF-κB	nuclear factor-κB
NO	nitric oxide
ER	endoplasmic reticulum
IBMIR	instant blood-mediated inflammatory reaction
ERKs	extracellular signal–regulated kinases
ATF-2	activating transcription factor-2
AP-1	activator protein-1
UW	University of Wisconsin solution
PFC	perfluorochemical
TLM	two-layer preservation method
ATP	adenosine triphosphate
EJ	extracellular-type/JNK inhibitor-containing solution
MKK	MAPK kinase
ICAM	intracellular adhesion molecule
APC	activated protein C
JIP1	JNK-interacting protein-1
IB1	islet-brain-1
JNKI	JNK inhibitory peptide
11R	11-arginine
